# Comparison of PED Flex and Lattice flow diverter in the treatment of unruptured intracranial aneurysms

**DOI:** 10.3389/fneur.2025.1535044

**Published:** 2025-03-14

**Authors:** Zhang Xuexian, Meng Xuerou, Ma Yuanjin, Xiong Bin, Pan Wenqiu, Zhao Wei, Wang Ruidong

**Affiliations:** ^1^Department of Interventional Medicine, The First Affiliated Hospital of Guangzhou Medical University, Guangzhou, China; ^2^Department of Intervention, The First Affiliated Hospital of Kunming Medical University, Kunming, China; ^3^Department of Interventional Medicine, The Second Affiliated Hospital of Wenzhou Medical University, Wenzhou, China; ^4^Department of Interventional Radiology, Qujing Second People’s Hospital, Qujing, China

**Keywords:** Pipeline Flex, Lattice, flow diverter, unruptured intracranial aneurysms, comparison

## Abstract

**Background and objective:**

Flow diverters (FDs) are widely used in the treatment of intracranial aneurysms (IAs). The Lattice flow diverter (LFD) is a novel FD developed in China, specifically designed for large or giant IAs. Currently, few studies have compared various FDs in the treatment of these conditions. This study endeavors to contrast the safety and efficacy of the Pipeline Flex embolization device (PED Flex) and LFD in the treatment of unruptured intracranial aneurysms (UIAs).

**Methods:**

This study retrospectively reviewed cases of UIAs managed with PED Flex or LFD at the Department of Interventional Radiology, Kunming Medical University’s First Affiliated Hospital from March 2022 to September 2024. We analyzed demographic characteristics, aneurysm features, medical history, complications, aneurysm occlusion, and clinical outcomes.

**Results:**

The study cohort consisted of 99 patients with 99 aneurysms, including 48 treated with PED Flex and 51 with LFD. The median follow-up duration was 9 months for both groups. Rates of complete aneurysm occlusion (81.3% vs. 78.4%, *p* = 0.727), successful aneurysm occlusion (87.5% vs. 86.3%, *p* = 0.857), and complication rates (2.1% vs. 3.9%, *p* = 0.727) did not differ significantly between the groups. Similarly, rates of in-stent stenosis (ISS) (14.6% vs. 11.8%, *p* = 0.678) and positive clinical outcomes were comparable.

**Conclusion:**

Our preliminary findings indicate that compared with PED Flex, the new domestic LFD has similar safety and effectiveness in treating UIAs. It is a new option for treating intracranial aneurysms and may have broad application prospects.

## Introduction

Intracranial aneurysm (IA) is a prevalent cerebrovascular disorder, with a global prevalence of 3 to 5% (1). Its rupture may lead to subarachnoid hemorrhage (SAH), a lethal form of stroke characterized by high mortality and disability rates. Approximately one-third of SAH patients will succumb to the condition, and survivors often suffer from severe neurological deficits. SAH, resulting from these ruptures, poses a significant health risk (2). Endovascular therapy has emerged as the preferred treatment modality for IA (3). Introduced by Medtronic in the United States and approved by the FDA in 2011, the Pipeline embolization device, a FD, was originally intended for large and giant wide-necked aneurysms within the internal carotid artery (ICA), ranging from the petrous to the supracavernous segment (4). FDs operate by altering the hemodynamics within the parent artery, significantly reducing blood flow velocity and pressure within the aneurysm, which promotes thrombosis. Over time, FDs endothelialize, leading to complete isolation and eventual occlusion of the aneurysm (5). The use of FDs has expanded from initially treating large aneurysms in the ICA to include posterior circulation aneurysms, as well as small and medium-sized aneurysms, yielding favorable outcomes (6–9).

The landscape of FD technology has rapidly evolved, yielding a variety of devices including the second-generation Pipeline Flex embolization device (PED Flex), PED Flex with Shield Technology (both from Covidien, United States), and additional devices like Surpass Streamline, Surpass Evolve (both from Stryker Neurovascular, United States), Flow Re-direction Endoluminal Device (FRED; MicroVention, United States), Silk FD (Balt Extrusion, France), and Tubridge FD (TFD, MicroPort, China) (10, 11). Notably, the PED Flex has been engineered to mitigate earlier challenges such as problematic distal release, inconsistent deployment, stent stenosis, migration, and issues with re-sheathing after partial deployment. Several studies have highlighted its enhanced clinical efficacy in aneurysm management (12–15). The Lattice flow diverter (LFD), a newer type of FD developed by ACCU Medical (Beijing, China), is a novel device designed to manage unruptured saccular or fusiform wide-necked aneurysms in the petrous segment to the terminal segment of the internal carotid artery (ICA) and the vertebral artery (VA), where the diameter of the parent artery is ≥2.0 mm and ≤ 5.6 mm. It is braided from 36 cobalt-chromium alloy wires (for radial support and wall adherence) and 12 platinum-tungsten wires (for full-length visualization). The metal coverage rate is 30–40%, the mesh density is 20–35/mm^2^, and the thickness of the stent body is 50 mm. It is currently the only FD whose delivery system comes with a mechanical balloon. In addition, both the stent and the mechanical balloon of this new FD adopt the MIROR (Metal interface Reassembly for Optimizing Restenosis) surface treatment process. The most significant difference between LFD and PED Flex is that the delivery system of LFD is equipped with a mechanical balloon. This feature facilitates the deployment of the stent and ensures its good adhesion to the vessel wall.

Comparative studies between PED Flex and LFD are currently limited. This paper aims to compare the safety and efficacy of PED Flex and LFD in patients with unruptured intracranial aneurysms (UIAs), and provide evidence-based guidance for neurointerventionists to select the most appropriate treatment device for patients with UIAs, thereby improving the overall treatment outcomes and quality of life of these patients.

## Materials and methods

### Study design

Data on UIAs managed with either PED Flex or LFD were gathered retrospectively from the Department of Interventional Medicine at the same institution for the specified period. The institution’s Ethics Committee sanctioned the study, exempting it from requiring informed consent due to its retrospective design.

Inclusion criteria: (1) patients aged between 18 and 80 years; (2) unruptured intracranial aneurysms (UIAs) treated with PED Flex or LFD; (3) IAs treated with a single flow diverter (FD). Exclusion criteria: (1) patients with aneurysms previously treated by endovascular methods or surgical clipping; (2) ruptured IAs; (3) incomplete data; (4) treatment of multiple aneurysms with a single FD; (5) concurrent cerebrovascular conditions such as arteriovenous fistula, arteriovenous malformation, or moyamoya disease.

### Antiplatelet therapy

Dual antiplatelet therapy was initiated 7 days prior to the procedure, consisting of aspirin (100 mg/day) and clopidogrel bisulfate (75 mg/day). Thromboelastography (TEG) was conducted 1 day before the procedure to assess the response to antiplatelet medications. Patients with a low response to clopidogrel were switched to ticagrelor (90 mg twice daily) alongside aspirin. Following the procedure, the initial regimen was continued for 6 months. If no ischemic symptoms or stent stenosis were observed upon reevaluation, patients were transitioned to lifelong monotherapy with aspirin (100 mg/day).

### Endovascular procedures

All interventions were performed under general anesthesia. An initial dose of heparin (50–70 U/kg) was administered based on patient weight at the start of the procedure, with an additional 1,000 U every hour. Following routine right femoral artery puncture, an 8F vascular sheath (Terumo, Japan) was inserted, and cerebral angiography was conducted using a 5F angiographic catheter (Terumo, Japan) to evaluate the aneurysm and plan treatment. A standard triaxial system was employed to access the target aneurysm: a 6F Neuron MAX long sheath (Penumbra, United States) was positioned at the parent artery’s origin, followed by a 5F or 6F Navien intermediate catheter (Medtronic, United States) guided to the proximal parent artery. Using two working angles from three-dimensional imaging, the aneurysm and parent artery were measured. The treatment involved guiding a Phenom 27 microcatheter (Medtronic, USA) or sine 27 microcatheter (ACCU, China) with a Synchro 14 microguidewire (Stryker, United States) across the aneurysm neck to the distal vessel for FD delivery and deployment. For FDs that failed to adhere properly, a microcatheter and microguidewire were used with a “massage” technique or, if necessary, a balloon catheter to ensure complete adherence to the artery wall.

### Data collection and follow-up

Patient case data were collected from the electronic medical record system, including demographic characteristics such as gender, age, symptoms at presentation (dizziness, aneurysm detection during physical examination), and history of hypertension, diabetes, and smoking. Details of the endovascular treatment recorded were aneurysm location, size, morphology, FD type, and use of coil-assisted embolization. Noted complications included postoperative cerebral infarction and SAH.

Patients were advised to undergo their first digital subtraction angiography (DSA) 3–6 months postoperatively, with subsequent DSAs every 6–12 months to assess aneurysm occlusion. The O’Kelly-Marotta (OKM) grading system was used to classify aneurysm filling status into four grades: grade A (completely filled, >95%), grade B (partially filled, 5–95%), grade C (residual neck filling, <5%), and grade D (no filling, 0%) (16). OKM grade D indicates complete occlusion, and OKM grades C and D are considered successful occlusion. The modified Rankin Scale (mRS) was employed to evaluate patient clinical status at discharge and at the last DSA, categorized into two levels: 0–2 (good outcome) and 3–6 (poor outcome) (17). ISS is defined as a stenosis exceeding 25% in a previously non-stenotic parent artery, with symptomatic ISS occurring in cases presenting with related ischemic symptoms (18).

### Statistical analyses

Statistical analyses were executed using SPSS version 25.0. Categorical data were reported as frequencies and percentages, and continuous data as means ± standard deviation (SD). The chi-square or Fisher’s exact test was employed for categorical variables, whereas continuous variables adhering to a normal distribution were assessed with the independent samples *t*-test, and those not normally distributed were analyzed using the Wilcoxon rank-sum test. Statistical significance was set at a *p*-value less than 0.05.

## Results

### Baseline characteristics

Following the study’s selection criteria, a total of 99 aneurysms from an equivalent number of patients were analyzed. Among these, 48 received treatment via PED Flex and 51 through LFD. The composition of the PED Flex cohort included 8 males and 40 females, with a mean age of 55.19 ± 10.15 years. Conversely, the LFD group was formed by 12 males and 39 females, with an average age of 56.10 ± 10.42 years.

In the PED Flex group, symptoms at presentation included headache (16 patients), dizziness (15), ptosis (1), and aneurysms discovered during physical examinations (16). The LFD group presented with headache (20), dizziness (11), ptosis (1), and discoveries during physical examinations (19). There were 19 hypertensive, 5 diabetic, and 5 smoking patients in the PED Flex group, compared to 23 hypertensive, 4 diabetic, and 10 smoking patients in the LFD group.

In terms of aneurysm location, the PED Flex group predominantly had aneurysms in the ICA, totaling 45, while none were observed in the middle cerebral artery and three were situated in the vertebrobasilar system. In the LFD cohort, 38 aneurysms were located in the ICA, one in the middle cerebral artery, and 12 in the vertebrobasilar artery. The average long diameters of the aneurysms were 6.98 ± 4.39 mm for the PED Flex group and 7.86 ± 5.33 mm for the LFD group, with neck lengths of 4.97 ± 3.46 mm and 6.38 ± 4.81 mm, respectively.

In the PED Flex group, 45 patients had saccular aneurysms, and 3 had non-saccular aneurysms, while in the LFD group, 38 had saccular and 13 had non-saccular aneurysms. Seven patients in each group underwent assisted coil embolization. The mRS scores at discharge were 0–2 for all patients in both groups.

No significant differences were found between the groups in terms of gender, age, symptoms, medical history, aneurysm size, and coil embolization (*p* > 0.05). However, significant differences in aneurysm location and morphology were noted (*p* < 0.05), with a higher proportion of vertebral-basilar artery dissecting aneurysms, predominantly non-saccular, in the LFD group.

**Figure 1:**
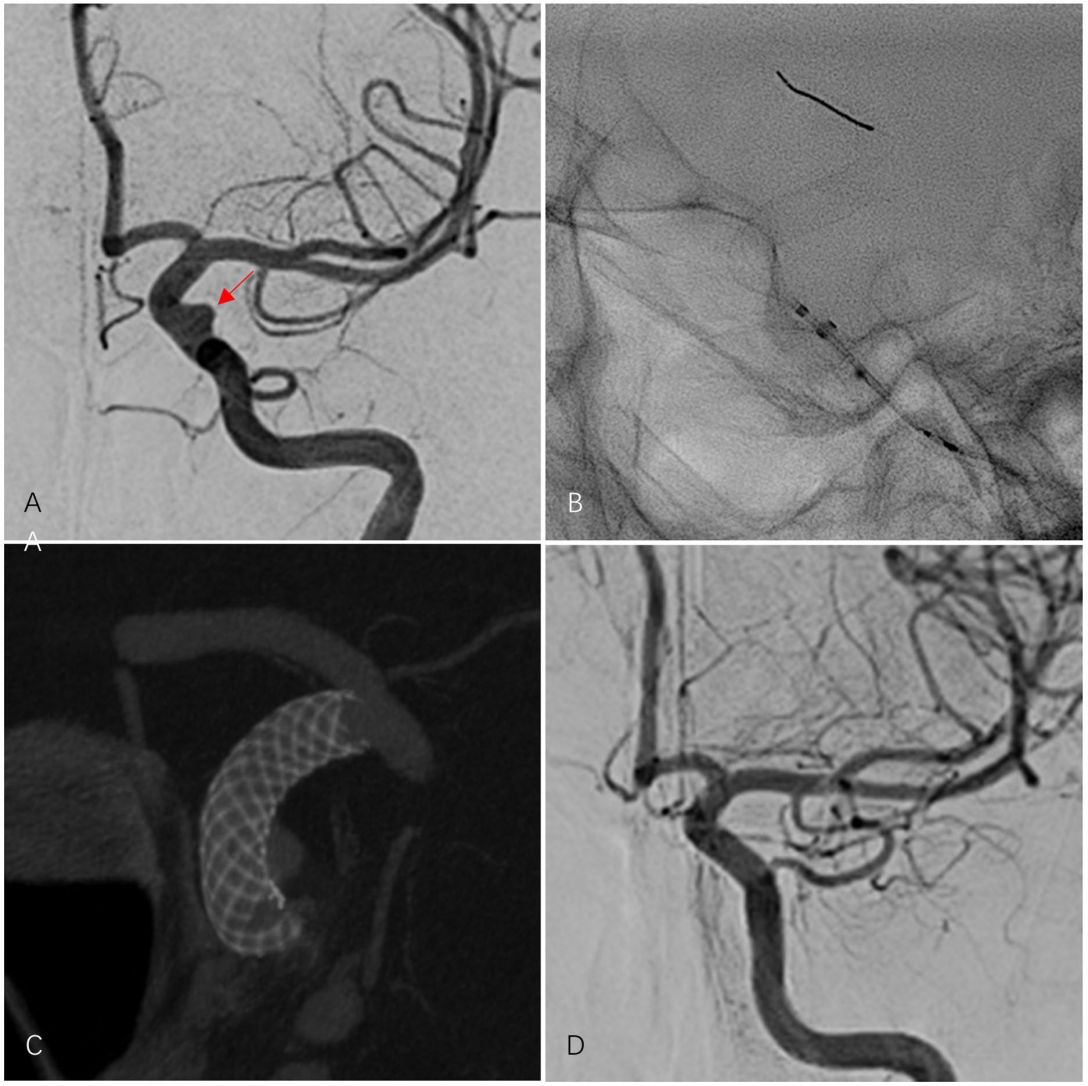
A 61-year-old female patient presented with an aneurysm in the C6 segment of the left internal carotid artery (ICA). The aneurysm was completely occluded after treatment with one PED Flex. **(A)** Pre-procedural DSA revealed the aneurysm in the left ICA C6 segment (indicated by a red arrow). **(B)** Controlled release of the PED Flex was performed. **(C)** Three-dimensional reconstruction after successful placement of the PED Flex confirmed that the stent was deployed and adhered well to the wall. **(D)** The final post-procedural angiographic follow-up demonstrated complete occlusion of the aneurysm (OKM grade D), a patent parent artery, and no ISS.

**Figure 2:**
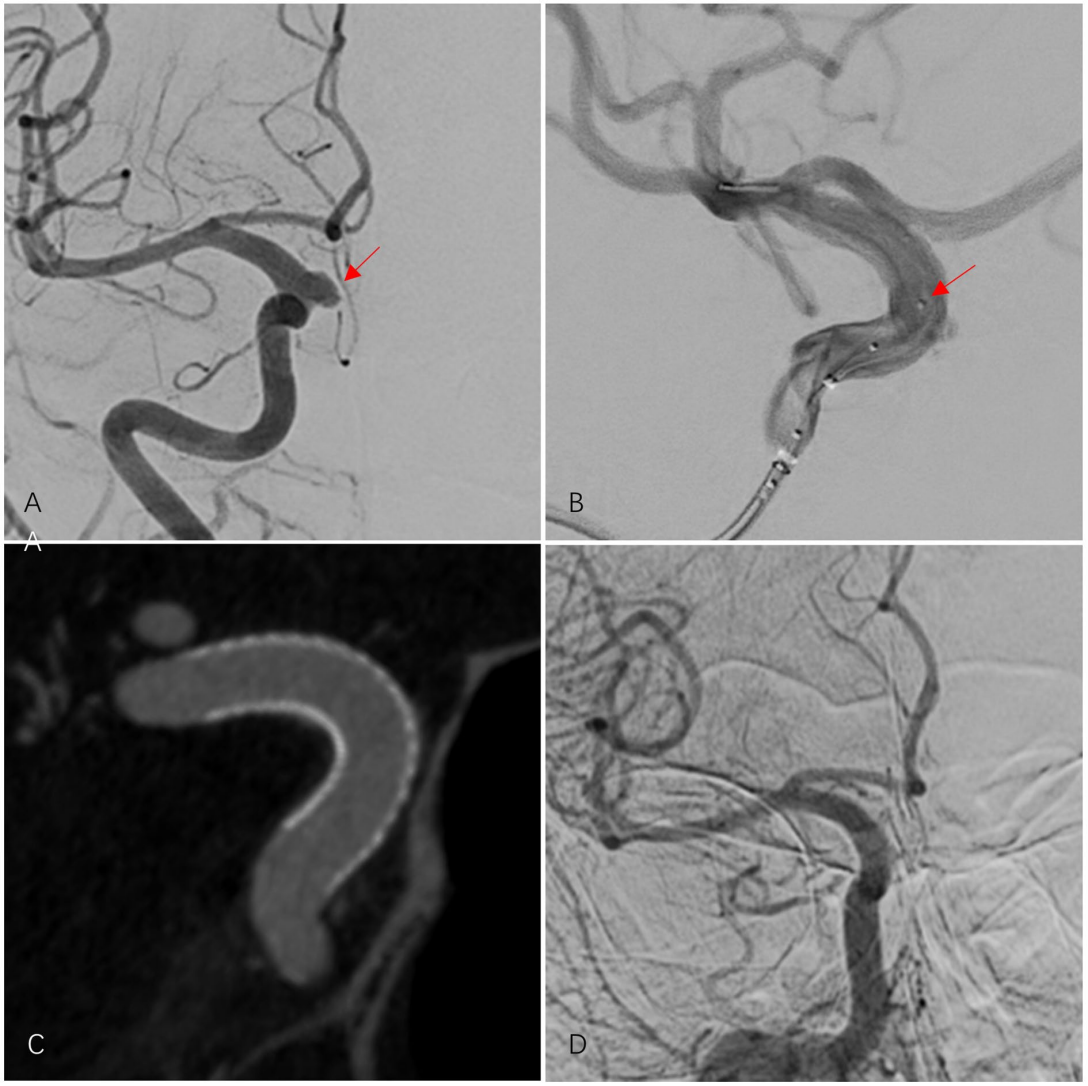
A 67-year-old female patient had an aneurysm in the C6 segment of the right internal carotid artery (ICA). After treatment with one LFD, the aneurysm was completely occluded. **(A)** Pre-procedural DSA shows the aneurysm in the right ICA C6 segment (indicated by a red arrow). **(B)** During the controlled release of the LFD, the metal spacing between mechanical balloons in the LFD delivery system is visible under DSA fluoroscopy (red arrow). **(C)** Three-dimensional reconstruction after successful placement of the LFD shows that the stent is deployed and adheres well to the wall. **(D)** The final angiographic follow-up after the procedure showed complete occlusion of the right ICA C6 segment aneurysm (OKM grade D), a patent parent artery, and no ISS.

Table 1 provides a detailed comparison of baseline data between the two groups. Figure 1 depicts a patient treated with PED Flex, and Figure 2 shows a patient treated with LFD.

**Table 1:** Demographic and endovascular treatment data comparison between groups.

Variable	PED Flex (*n* = 48)	LFD (*n* = 51)	*p*-value
Demographic characteristics			
**Gender**			
Male	8	12	0.395
Female	40	39	
Age, years	55.19 ± 10.15	56.10 ± 10.42	0.661
**Symptom**			
Headache	16	20	0.543
Dizziness	15	11	0.274
Drooping eyelids	1	1	1.000
Physical examination findings	16	19	0.683
**Past medical history**			
Hypertension	19	23	0.579
Diabetes	5	4	0.924
Smoking	5	10	0.202
Endovascular treatment details			
**Aneurysm location**			
ICA	45	38	0.009
Middle cerebral artery	0	1	1.000
Vertebrobasilar artery	3	12	0.017
Aneurysm long diameter (mm)	6.98 ± 4.39	7.86 ± 5.33	0.375
Aneurysm neck length (mm)	4.97 ± 3.46	6.38 ± 4.81	0.096
**Aneurysm morphology**			
Saccular	45	38	0.020
Non-saccular	3	13	
**Additional coil embolization**			
Yes	7	7	0.903
No	41	44	
**mRS score at discharge**			
0–2	48	51	—
3–6	0	0	

### Follow-up outcomes

Both treatment groups, PED Flex and LFD, were followed angiographically for a median period of nine months. The rates of complete aneurysm occlusion were 81.3% for the PED Flex group and 78.4% for the LFD group, with no statistically significant difference noted (*p* > 0.05). The rates of successful occlusion stood at 87.5% in the PED Flex group and 86.3% in the LFD group, also without a significant difference (*p* > 0.05). Although differences in complete and successful occlusion rates were not statistically significant, they were slightly higher in the PED Flex group compared to the LFD group.

The incidence of post-procedure ISS was 14.6% in the PED Flex group and 11.8% in the LFD group, with no significant difference noted (*p* > 0.05). No ischemic symptoms were reported in cases of ISS in either group. Post-procedure cerebral infarctions occurred in one patient in the PED Flex group and two patients in the LFD group; however, all affected patients had an mRS score of 0–2 at the last follow-up. No cases of SAH were reported post-procedure in either group. The rate of good prognosis (mRS score of 0–2) at the last follow-up was 100% for both groups. The detailed follow-up out-comes of the two groups are shown in Table 2.

**Table 2:** Angiographic and clinical follow-up data comparison between groups.

Variable	PED Flex	LFD	OR	*p*-value
Aneurysm occlusion status at the last angiographic follow-up				
Complete occlusion (*n*, %)	39 (81.3%)	40 (78.4%)	1.19	0.727
Successful occlusion (*n*, %)	42 (87.5%)	44 (86.3%)	1.11	0.857
Post-procedure ISS	7 (14.6%)	6 (11.8%)	1.28	0.678
Symptomatic ISS	0	0	—	—
Complication				
Post-procedure cerebral infarction	1 (2.1%)	2 (3.9%)	0.52	1.000
Post-procedure SAH	0	0	—	—
Follow-up time (months)	9 (3, 15)	9 (3, 12)	1.08	0.931
mRS score at the last clinical follow-up				
0–2	48	51	—	—
3–6	0	0	—	

## Discussion

With the rapid advancement of neurointerventional technologies, an increasing array of new FDs is being adopted in leading clinical centers. Despite their growing use, these new FDs could introduce unforeseen risks. While the underlying principles and mechanisms of FDs in treating IAs are similar, variations in design and materials may influence patient outcomes. It is crucial to assess the safety and efficacy of different FDs. LFD is the first FD featuring a mechanical balloon. The mechanical balloon has the following advantages. Firstly, the mechanical balloon is equipped with multiple radiopaque markers. Its first marker is aligned with the landing point of the stent, which helps with the positioning of the stent and may reduce the damage to the blood vessel caused by the stent dragging. Moreover, during the deployment process, its multiple radiopaque markers can assist in judging the progress of the stent deployment. Secondly, the mechanical balloon can help with the deployment of the stent to ensure good adherence to the vessel wall. Finally, due to the design of the mechanical balloon, the tip of the delivery guidewire will be relatively stable, reducing the possibility of the delivery guidewire suddenly moving forward and damaging the blood vessel. However, precisely because of the existence of the mechanical balloon, the delivery system of this FD tends to be rather rigid, which may lead to difficulties when it is delivered through very tortuous blood vessels and may even make it impossible to deliver it to the parent artery of the aneurysm. Additionally, the LFD stent uses MIROR surface treatment process, which may be beneficial for reducing the formation of thrombus during and after the operation, accelerating the endothelialization process, and may also reduce the incidence of long-term in-stent stenosis.

Our study contrasted outcomes from the long-established PED Flex with the newly introduced Chinese-made LFD. Results indicated that short-term outcomes between the two devices were comparable. No significant differences were observed in complete occlusion rates (81.3% vs. 78.4%, *p* = 0.727), successful occlusion rates (87.5% vs. 86.3%, *p* = 0.857), complication rates (2.1% vs. 3.9%, *p* = 0.857), rates of ISS (14.6% vs. 11.8%, *p* = 0.678), or rates of good prognosis.

### Complete occlusion rate compared with previous studies of FD for UIAs

The complete occlusion rate of aneurysms treated with PED Flex in our study is comparable to that in several prior studies (14, 15, 17, 18). A recent meta-analysis on the TFD reported a complete occlusion rate of 78% with a mean follow-up of less than 12 months (19). Another meta-analysis concerning the Derivo embolization device, which included 481 aneurysms, found a complete occlusion rate of 81.4% over a 9–18 month follow-up period (20). Vivanco-Suarez et al. (21) found that the Surpass Evolve had a complete occlusion rate of 73% at a median follow-up of 10.2 months. In our study, the LFD achieved a complete occlusion rate of 78.4% at a median follow-up of 9 months, demonstrating satisfactory performance. In our study, the aneurysm occlusion rate in the PED Flex group was slightly higher than that in the LFD group. However, the difference between the two groups was not statistically significant. This might be related to the single-center nature of our study and the small sample size. It could also be associated with the differences in the location and morphology of aneurysms between the two groups. In addition, we also observed a relatively high proportion of patients with vertebral-basilar artery dissecting aneurysms in the LFD group. This may suggest that LFD remains highly effective in treating posterior circulation aneurysms or dissecting arteries. Of course, this view requires further research for verification.

### Complications compared with previous studies of FD for UIAs

Li et al. (22) reported a complication rate of 2.5% when using PED Flex to treat IAs, with no patient deaths. Chen et al. (14) noted a complication rate of 3.1% for PED Flex, including ischemic complications in 2.3% and hemorrhagic complications in 0.8%, with a good prognosis rate (mRS 0–2) of 99.2%. In our study, the PED Flex group had a complication rate of 2.1%, with no hemorrhagic complications and a good outcome rate of 100%, aligning closely with these findings. Monteiro et al. (20) found that the Derivo embolization device had a complication rate of 4.9% for ischemic and hemorrhagic complications. A systematic review of Surpass Evolve reported a neurological complication incidence of 6.2%, with most patients experiencing mild symptoms (23). A multicenter retrospective study on the TFD documented neurological complications in 5.4% of cases, including cerebral infarctions in 4.2%, with 93.2% achieving a good prognosis (24). In our study, the LFD group had a complication rate of 3.9%, with no hemorrhagic complications, and maintained a good prognosis rate of 100%, which is consistent with the results of newer FD studies.

### ISS compared with previous FD studies for the treatment of UIAs

El Naamani et al. (17) reported an ISS rate of 14.2% for PED/PED Flex and 14.6% for PED Shield in treating IAs. Huang et al. (18) found that the rates were 17.1% with PED and 16.6% with TFDs. Han et al. (25) observed an ISS incidence of 17.0% across FD treatments. In our study, the ISS rates were 14.6% in the PED Flex group and 11.8% in the LFD group, comparable to these findings, with all cases being asymptomatic.

### Limitations

This study adopts a single-center, retrospective design, which is characterized by regional limitations. Moreover, the small sample size of this study may lead to selection bias, both of which restrict the generalizability of the research results. Additionally, the follow-up duration was insufficient to observe long-term outcomes of aneurysm occlusion and ISS. Future studies should be multicenter and prospective with larger sample sizes to validate our findings.

## Conclusion

Our preliminary research results indicate that PED Flex and LFD are comparable in terms of safety and efficacy in the treatment of UIAs, potentially providing more treatment options for clinicians. However, due to the limitations of this study, prospective, large sample, multicenter studies are needed in the future to further validate this conclusion.

## Data Availability

The raw data supporting the conclusions of this article will be made available by the authors, without undue reservation.
